# Allele‐specific proximal promoter hypomethylation of the telomerase reverse transcriptase gene (*TERT*) associates with *TERT* expression in multiple cancers

**DOI:** 10.1002/1878-0261.12786

**Published:** 2020-09-11

**Authors:** Teisha J. Rowland, Andrew J. Bonham, Thomas R. Cech

**Affiliations:** ^1^ Department of Biochemistry BioFrontiers Institute University of Colorado Boulder Boulder CO USA; ^2^ Howard Hughes Medical Institute University of Colorado Boulder Boulder CO USA; ^3^ Department of Chemistry & Biochemistry Metropolitan State University of Denver Denver CO USA

**Keywords:** epigenetics, monoallelic gene expression, promoter CpG methylation, telomerase reverse transcriptase, *TERT*

## Abstract

Telomerase reverse transcriptase (TERT) is pathologically expressed in the vast majority of human cancers, but the epigenetic regulation of its expression is only beginning to be understood. In particular, the active *TERT* gene in cancer cells has been characterized as having a hypermethylated CpG island, opposite to the general association of DNA methylation with gene repression. Here, we analyzed *TERT* promoter CpG methylation in 833 human cancer cell lines representing 23 different tissue types and found hypermethylation of the upstream portion of the CpG island and more conserved hypomethylation of a region including the proximal *TERT* promoter and exon 1. In cell lines with monoallelic expression of *TERT*, we found allelic methylation of the proximal *TERT* promoter. This included cell lines with the −124 or −146 activating promoter mutation as well as wild‐type *TERT* cancer lines. In these cell line types, decreased proximal promoter methylation is associated with the active allele. Compared to cells with monoallelic expression of *TERT*, lines with biallelic expression of *TERT* had even lower methylation in the proximal *TERT* promoter. Thus, in cell lines from cancers of many different tissues, the *TERT* proximal promoter has canonical DNA methylation, with low methylation correlating with increased *TERT* expression.

## Graphical abstract

In cancer, active expression of the *TERT* gene paradoxically correlates with a hypermethylated CpG island. Here, we show that in 833 cancer cell lines representing 23 tissue types, the proximal promoter contains conserved hypomethylation. In lines with monoallelic *TERT* expression, decreased proximal promoter methylation associates with the active allele. Thus, the proximal *TERT* promoter has canonical DNA methylation.

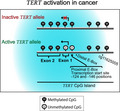

**Abbreviations**
BAE: biallelic expression
CCLE: Cancer Cell Line Encyclopedia
CGI: CpG island
ChIP‐Bis‐Seq: ChIP–bisulfite conversion sequencing
CpG: cytosine–guanine sequence
ETS: E‐twenty‐six
FISH: fluorescence innonbreakingspacesitu hybridization
GABP: GA‐binding protein
gDNA: genomic DNA
H3ac: histone H3 acetylation
MAE: monoallelic expression
SNP: single‐nucleotide polymorphism
TERT: telomerase reverse transcriptase
TF: transcription factor
WT: wild‐type

## 1 Introduction

Serving as protective caps at the ends of eukaryotic chromosomes, telomeres maintain chromosomal and genome stability. Telomeric DNA can be lengthened by telomerase, a ribonucleoprotein enzyme [1, 2]. While telomerase is expressed during normal human development, it becomes inactive in most somatic cells. Telomeres then progressively shorten due to the ‘end replication problem’ until they reach a critical length, and the aged cells undergo senescence. However, in most malignant human cancers (≈ 80–90%), telomerase is pathologically active, allowing cellular immortalization [3, 4, 5]. The expression of the catalytic subunit of telomerase, TERT [6], is limiting for telomerase in most cells, because the RNA subunit (hTR) is constitutively present. Indeed, some human cells can be immortalized simply by ectopic expression of TERT [7, 8].

In ≈ 22% of cancer cell lines, *TERT* reactivation is clearly genetic, occurring through activating promoter mutations [9]. Two common *TERT‐*activating mutations are C>T transitions located −124 and −146 bp upstream of the *TERT* translation start codon (AUG) (chr5:1295228 and chr5:1295250, or C228T and C250T, respectively; hg19 genomic coordinates). These mutations create a new binding site for E‐twenty‐six (ETS) transcription factors and recruit the GA‐binding protein (GABP) [2, 10]. These mutations are heterozygous, and only the promoter‐mutant *TERT* allele is active, resulting in monoallelic expression (MAE) of *TERT*. An alternative explanation for MAE of *TERT* is that the promoter mutation abrogates silencing of that allele [11]. Although this pathway is distinct from the reactivation model, the end result is the same: The promoter‐mutant *TERT* allele is transcriptionally active, while the other alleles of *TERT* are epigenetically silenced.

*TERT* reactivation can also be entirely epigenetic, and this appears to be the case for the majority of cancers. Approximately 71% of these *TERT* WT cell lines show biallelic expression (BAE) of *TERT* [9]. Intriguingly, cancer cell lines with wild‐type (WT) *TERT* sequences, containing no known activating *cis*‐acting genetic alterations, sometimes show MAE [9, 12, 13]. These MAE vs. BAE line classifications are based on expression of exonic *TERT* single‐nucleotide polymorphisms (SNPs). However, using single‐cell fluorescence *in situ* hybridization (FISH) imaging, we recently found that there is actually considerable line‐to‐line (and cell‐to‐cell) heterogeneity in the number of *TERT* transcription sites and gene copies, and in the ratio of the two [14]. In other words, while MAE cell lines express only one version of the *TERT* gene and BAE lines express multiple versions, the ratio of active to inactive *TERT* gene copies is not simply 1 : 1 and 2 : 0 in MAE and BAE lines, respectively. Regardless of ‘MAE’ or ‘BAE’ classification, all lines have inactive copies as well as one or multiple active WT *TERT* gene copies. It is important to understand the epigenetic mechanisms reactivating these copies (or failing to silence them). Epigenetic activation involves histone modifications [15] and DNA methylation, described next in this section.

In mammalian genomes, DNA CpG methylation impacts gene transcription, though not always in a straightforward manner. CpG sites contain a methylated cytosine ≈ 80% of the time [16]. This methylation affects chromatin structure and binding of transcription‐associated factors. Genome‐wide demethylation is an early cancer hallmark [17]. This primarily manifests as demethylation of intergenic and highly repeated sequences, possibly causing activation of noncoding RNAs [16, 17, 18]. Hypomethylation of promoters is also apparent, which correlates with overexpression of oncogenes and other genes associated with tumor invasion or metastasis. Intriguingly, cancer‐associated hypermethylation of promoters in CpG islands (CGIs; GC‐rich regions typically spanning ≈ 1 kb) represses transcription of tumor suppressors [16, 17, 18, 19, 20]. In healthy adult somatic cells, promoters in CGIs are typically unmethylated and associated with active gene expression [16].

Because promoter hypermethylation canonically associates with repressed transcription, the *TERT* promoter has appeared noncanonical, though this may be due to a focus on the upstream promoter region. The relatively large *TERT* promoter CGI spans ≈ 4 kb, approximately −1800 to +2200 bp relative to the *TERT* AUG (chr5:1295228, hg19). It is extremely GC‐rich, possessing up to 70% GC content [16, 21]. Paradoxically, the *TERT* promoter CGI is primarily hypomethylated and inactive in healthy adult somatic cells [22], yet hypermethylated and active in cancer cells. In the upstream *TERT* promoter, hypermethylation of a CpG (cg11625995, −628 of the AUG) has been used as a reliable biomarker for *TERT* expression, tumor progression, and prognosis [23]. Methylation of repressor binding sites here may lead to *TERT* reactivation [16, 24]. However, because this region is hypomethylated in pluripotent cells that actively express TERT [22, 25], this explanation is insufficient. Additionally, decreased methylation in this upstream region actually associates with active transcription histone marks [26].

A limited hypomethylated region flanking the *TERT* transcription start site region may serve as a ‘minimal promoter’. This region roughly spans −200 to +100 bp of the transcription start site or −260 to +40 bp of the AUG [27]. This hypomethylation is consistent between both WT and *TERT* promoter‐mutant cancer cell lines [26]. Within it are multiple methylation‐sensitive transcription factor (TF) binding sites. These include two E‐Box sites (−236 and −28 bp of the AUG) that repressive Mad/Max or activating c‐Myc/Max TFs bind [16, 24, 28, 29] (Table S1) (for a detailed description of *TERT* promoter TF binding sites, see Ref. [24]). Active chromatin marks have been associated with unmethylated DNA in this region [16, 27].

Better understanding of *TERT* promoter methylation patterns may enable us to decipher the different mechanisms by which *TERT* is reactivated in different cancer types. For example, *TERT‐*activating mutations are more prevalent in some types of cancers (e.g., melanoma and medulloblastoma) [9]. These possess allelic methylation in the hypermethylated, upstream promoter region, where decreased methylation associates with the active allele [26]. Allelic methylation in the minimal promoter of mutant cells has only been reported in thyroid cancer cell lines [30], but its more general occurrence and its presence in WT lines, with either MAE or BAE of *TERT*, are unknown. Additionally, while WT MAE and BAE lines associate with different cancer types [9]—for example, pancreatic cancers are frequently WT MAE, while lung cancers are primarily WT BAE—it has been unknown whether there are associated *TERT* promoter methylation patterns.

Here, we investigated DNA methylation patterns in the *TERT* promoter across 23 different cancer tissue types and 833 different cancer cell lines. In all cancerous tissue types, we found hypermethylation in the upstream promoter region and hypomethylation of a proximal promoter region and exon 1. The proximal promoter hypomethylation appeared more conserved across different tissue types than the upstream hypermethylation, which varied significantly between different tissues. Within the hypomethylated proximal promoter, apparently BAE lines had significantly decreased methylation relative to MAE WT and mutant lines, suggesting decreased methylation here to be important for increased *TERT* transcription. This region also contained allelic methylation in MAE lines, with decreased methylation associating with active transcription. Overall, it appears that hypomethylation of the *TERT* proximal promoter is important for *TERT* expression in cancer cells.

## 2 Materials and methods

### 2.1 CCLE data analysis

Cancer cell line DNA CpG methylation data were downloaded from the Broad Institute's Cancer Cell Line Encyclopedia (CCLE) [31] (www.broadinstitute.org/ccle, June 14, 2018, release, *TERT* gene) for the *TERT* gene and 1 kb upstream of the translation start site. Data were visualized using python (Python Software Foundation, Beaverton, OR, USA) with the libraries plotly [32] and pandas (NumFOCUS, Austin, TX, USA). Full CCLE dataset includes data from 833 cancer cell lines with bisulfite conversion sequencing (Bis‐Seq) CpG methylation data at 224 genomic positions. For Fig. 2A, raw read Bis‐Seq DNA CpG methylation data from CCLE's Bis‐Seq dataset were obtained by direct request to the Broad Institute's CCLE and analyzed for allelic patterns using stringent cutoff criteria (reads analyzed contained 3–6 CpGs per read, and coverage of ≥ 5 reads per cell line). Dataset BAM files were analyzed using python with pysam [33] and scipy libraries [34], as well as the methylation analysis tool ‘quma’ (http://quma.cdb.riken.jp/) [35]. Graphs were prepared using prism 8 (GraphPad, San Diego, CA, USA, version 8.4.0).

### 2.2 ENCODE UCSC Genome Browser data analysis

Cell line data for normal, cancer, and human embryonic stem cell (hESC) line DNA CpG methylation data (Fig. S2 only) were downloaded as BED files from the Encyclopedia of DNA Elements (ENCODE) at UC Santa Cruz (UCSC) Genome Browser for the *TERT* gene and 1 kb upstream of the translation start site [36]. Data were visualized using python (Python Software Foundation) with the libraries plotly [32] and pandas (NumFOCUS).

### 2.3 Cell lines and culture

Lines DB, NCI‐H196, and RPMI 8226 [American Type Culture Collection (ATCC), Manassas, VA, USA] were maintained in RPMI‐1640 medium (Gibco Thermo Fisher Scientific, Waltham, MA, USA). Lines U‐87 MG [University of Colorado Cancer Center, Protein Production/MoAB/Tissue Culture Shared Resource (PPSR)] and LN‐18 (ATCC) were maintained in Dulbecco's modified Eagle's medium (DMEM) (Gibco Thermo Fisher Scientific). SK‐N‐SH (PPSR) and adult human foreskin fibroblasts were maintained in Eagle's minimum essential medium (EMEM) (Gibco Thermo Fisher Scientific). All media were supplemented with 100 μg·mL^−1^ penicillin and 100 μg·mL^−1^ streptomycin (Gibco Thermo Fisher Scientific) and 10% (Sigma‐Aldrich, St. Louis, MO, USA) or 5% (only line LN‐18) fetal bovine serum (FBS) (Peak Serum Inc., Wellington, Colorado, US). All lines were cultured according to recommended protocols.

### 2.4 DNA isolation, PCR, and sequencing

DNA isolation, PCR, and Sanger sequencing (GENEWIZ, South Plainfield, NJ, USA) were performed as previously described [14]. Briefly, gDNA was isolated from cells using Quick‐DNA Miniprep Kit (11‐317AC; Zymo Research, Irvine, CA, USA). PCRs (20 μL) were performed using 50 ng of gDNA and Phusion High‐Fidelity DNA Polymerase (F‐530; Thermo Fisher Scientific, Grand Island, NY, USA) supplemented with 7‐deaza‐2′‐deoxy‐guanosine‐5′‐triphosphate (7‐Deaza‐dGTP) (10988537001; Sigma‐Aldrich) to aid in amplifying GC‐rich regions. Sequences for primers (Integrated DNA Technologies, Coralville, IA, USA) are listed in Table S4. PCR products were purified using E.Z.N.A. Cycle Pure Kit (D6492; Omega Bio‐Tek).

### 2.5 RNA extraction, cDNA synthesis, and RT‐PCR

RNA extraction, cDNA synthesis, and RT‐PCR were performed as previously described [14]. Briefly, total RNA was isolated using the E.Z.N.A. Total RNA Kit I (R6834; Omega Bio‐Tek, Norcross, GA, USA) and RNase‐free DNase Set I (E1091‐02; Omega Bio‐Tek). RNA (1 μg) was used to synthesize cDNA with the SuperScript IV First‐Strand Synthesis System (Invitrogen Thermo Fisher Scientific, Waltham, MA, USA; 18091050). RT‐PCR was performed with SYBR Select Master Mix (4472908; Thermo Fisher Scientific) supplemented with 7‐Deaza‐dGTP using the lightcycler 480 software (Roche, Basel, Switzerland). Primers used were previously described except for TERT exon 2 primers (primer sequences and citations are listed in Table S4). RT‐PCRs (10 μL) were run in triplicate on a 96‐well plate and data normalized to the geometric mean of 3 ‘housekeeping’ genes [glucose phosphate isomerase (GPI), peptidylprolyl isomerase A (PPIA), and hydroxymethylbilane synthase (HMBS)] [14]. PCR products were purified using E.Z.N.A. Cycle Pure Kit (D6492; Omega Bio‐Tek) and underwent Sanger sequencing (GENEWIZ).

### 2.6 Bisulfite conversion cloning

For Fig. 2B, gDNA was isolated from cells using the Quick‐DNA Miniprep Kit (11‐317AC; Zymo Research) and 300 ng underwent bisulfite conversion using the EZ DNA Methylation‐Gold Kit (D5005; Zymo Research). Twenty nanogram of bisulfite‐converted DNA was used in 25 μL PCR amplification reactions with primers flanking the *TERT* proximal promoter (5:1295138–1295413; 33 CpGs included; 331 bp PCR product), 1.25 units of EpiMark Hot Start Taq DNA Polymerase [M0490; New England BioLabs (NEB), Ipswich, MA, USA], and the following thermocycling conditions: 95 °C for 30 s, then 40 cycles of 95 °C for 30 s, 55 °C for 60 s, and 68 °C for 30 s, followed by a final extension of 68 °C for 5 min. Primers used were modified from a previous publication [22] which had designed primers to complement and amplify methylated CpGs in hypomethylated cell types. Here, in initial experiments we found that these primers led to unrepresentative overamplification of methylated CpGs in cancer cells. Hence, we modified these primers to complement unmethylated CpGs; sequences for primers (Integrated DNA Technologies) are listed in Table S4. To prepare PCR products for blunt‐end cloning, 5′‐end phosphorylation was performed using T4 polynucleotide kinase (M0201; NEB) and 3′ overhang removal using T4 DNA polymerase (M0203; NEB) and purified using E.Z.N.A. Cycle Pure Kit (D6492; Omega Bio‐Tek). To prepare the vector, 1 μg pUC19 DNA was digested using SmaI restriction enzyme (R01415; NEB), treated with alkaline phosphatase (M0290; NEB), and purified using E.Z.N.A. Cycle Pure Kit. Prepared PCR products were ligated into the vector using Quick Ligation (M2200; NEB). Supercompetent cells were transformed and incubated on Carb LB agar plates overnight at 37 °C, colonies were picked and incubated in 5 mL LB overnight at 37 °C while shaking, plasmids were purified using E.Z.N.A. Plasmid Mini Kit I (D6942; Omega Bio‐Tek), and inserts were sequenced using Sanger sequencing. All cloning details were according to recommended manufacturer's protocols. Methylation sites were visualized, and quality control was performed using the online tool ‘quma’ (http://quma.cdb.riken.jp/) [35].

### 2.7 Long‐range bisulfite conversion PCR

For Fig. 3B,C, long‐range bisulfite conversion PCR was optimized following previously published guidelines for generating large bisulfite‐converted PCR products [37]. gDNA was isolated from cells using Quick‐DNA EZ DNA Methylation‐Gold Kit and underwent bisulfite conversion using the Methylamp DNA Modification Kit (P‐1001; EpiGentek, Farmingdale, NY, USA). A two‐step PCR amplification was used: For the first step, 50 ng of bisulfite‐converted DNA was used in 20 μL reactions with 3.5 units of EpiMark Hot Start Taq DNA Polymerase and the following thermocycling conditions: 94 °C for 2 min, then 35 cycles of 94 °C for 20 s, 62 °C for 45 s, and 65 °C for 1 min and 55 s, followed by a final extension of 65 °C for 5 min; for the second step, 1 μL of a 1 : 50 dilution of the first‐step PCR product was used in 25 μL reactions with 3.5 units of EpiMark Hot Start Taq DNA Polymerase and the same thermocycling conditions as the first step except the 62 °C annealing temperature was increased to 67 °C. PCR products were validated on an agarose gel, gel‐purified using the MinElute Gel Extraction Kit (28606; Qiagen, Hilden, Germany), and sequenced using Sanger sequencing. Unmethylated‐ and methylated‐specific primers in the *TERT* promoter upstream of the *TERT* translation start site contained three CpGs to amplify unmethylated or methylated DNA by containing a C (to complement methylated DNA) or T (to complement unmethylated DNA) within the primers at all three CpGs. For both of these promoter primers, the same reverse primer was used, which was downstream of the exon 2 SNP in a methylated region. The bisulfite conversion PCR generated a relatively large (1448 bp) PCR product, which was sequenced using Sanger sequencing. Sequences for primers (Integrated DNA Technologies) are listed in Table S4.

### 2.8 ChIP‐Bis‐Seq library construction and data analysis

ChIP was performed as previously described [15, 26] with modifications. LN‐18 cells were cultured until approximately 80% confluent, rinsed with PBS, fixed with freshly prepared 1% (v/v) formaldehyde (BP531500; Fisher Scientific) in PBS at room temperature (RT) for 10 min, and inactivated using 1.25 m glycine solution for 2 min at RT. Following solution aspiration, cells were scraped and collected, resuspended in ice‐cold PBS, and centrifuged at 1000 ***g*** at 4 °C for 5 min. The resultant pellet was frozen at −80 °C for at least 60 min and then lysed for 10 min on ice in 300 μL lysis buffer (50 mm Tris/Cl pH 8.1, 10 mm EDTA, 0.5% SDS) with 6 μL 50× protease inhibitor (A32965; Thermo Fisher Pierce, Waltham, MA, USA) added immediately prior to use. Chromatin was sonicated using a BioRuptor for 4 × 10 min on ‘high’, for 30 s ‘on’ and 30 s ‘off’, in ice water, resulting in fragmented pieces of approximately 200–500 bp, as confirmed using a purified sample via gel electrophoresis on a 1% agarose gel. Following sonication, chromatin was initially cleared via centrifugation at 16 000 ***g*** for 15 min at 4 °C and supernatant transferred to a new tube and quantified via NanoDrop. Next, 20 μg of cleared chromatin (15–50 μL per sample) was further cleared to reduce background via nutation for 2 h at 4 °C in 1–2 mL immunoprecipitation (IP) buffer (16.7 mm Tris/Cl pH 8.1, 1.2 mm EDTA, 167 mm NaCl, 1% Triton X‐100) with 50–100 μL A/G magnetic beads (88803; Pierce Protein, Waltham, MA, USA) that had been washed twice in IP buffer. Using a magnetic rack, cleared chromatin was recovered in 500 μL IP buffer and incubated with 5 μg anti‐H3ac antibody (06‐599; Millipore, Burlington, MA, USA) or 5 μg rbt IgG (12‐370; Millipore) overnight with nutation at 4 °C. Immunoprecipitation was performed using 25 μL washed A/G magnetic beads for 1 h with nutation at RT. Following immunoprecipitation, IP buffer was removed and nonspecific binding reduced by washing the beads in 1 mL low‐salt buffer (20 mm Tris/Cl pH 8.0, 2 mm EDTA, 150 mm NaCl, 0.1% SDS, 1% Triton X‐100), high‐salt buffer (20 mm Tris/Cl pH 8.0, 2 mm EDTA, 500 mm NaCl, 0.1% SDS, 1% Triton X‐100), and lithium chloride (LiCl) buffer (10 mm Tris/Cl pH 8.0, 1 mm EDTA, 250 mm LiCl, 1% sodium deoxycholate, 1% IGEPAL), and then washed in Tris/EDTA (TE) (20 mm Tris pH 8, 2 mm EDTA) to remove salts. The antibody:IP complex was eluted in 120 μL elution buffer (100 mm NaHCO_3_, 1% SDS, 200 mm NaCl) at RT with mixing for 20 min. During elution, an input sample was included that had been frozen immediately following the first clearing step. For crosslink reversal, supernatant was incubated at 65 °C for > 6 h. Protein and RNA were removed through incubation with 100 mm Tris pH 6.5, 11 mm EDTA, 60 μg Proteinase K (AM2544; Life Technologies, Carlsbad, CA, USA), and 1 μg DNase‐free RNase (EN0531; Thermo Scientific) for 60 min at 37 °C. DNA was purified using E.Z.N.A. Cycle Pure Kit (D6492; Omega Bio‐Tek), resuspended in TE, and underwent RT‐PCR as described in Section 2.5 above.

For ChIP‐Bis‐Seq library construction, ChIP was performed using biological duplicates. Duplicate pull‐down and input samples were used for library construction. Fifty nanogram of pulled‐down ChIP DNA was used with the NuGEN Ovation Ultralow Methyl‐Seq DR Multiplex System (0335‐32; NuGEN, Tecan Genomics, Inc., Redwood City, CA, USA), according to the manufacturer's instructions. DNA was assessed using a Qubit Fluorometer 3.0 (Thermo Fisher Scientific). Sonicated, pulled‐down gDNA fragments underwent end repair, ligation of kit‐provided methylated adaptors, and final repair, following the manufacturer's instructions. Eluted library samples underwent qPCR to determine the number (*N*) of PCR cycles required for library amplification (QuantStudio 6 Real‐Time PCR; Thermo Fisher Scientific). Amplified libraries were purified with Agencourt beads and eluted in low‐EDTA TE buffer. Bioanalyzer 2100 (Agilent, Santa Clara, CA, USA) was used to validate and quantify libraries. Amplified libraries were normalized and pooled, denatured, and diluted for sequencing on MiSeq (Illumina, San Diego, CA, USA) according to manufacturer's guidelines.

For ChIP‐Bis‐Seq data analysis, prior to alignment, single‐end reads were filtered using FastQC (Babraham Institute, Cambridge, UK) and adaptor‐trimmed using cutadapt [38]. Only reads with a *Q* score ≥ 20 and matching length criteria were used for mapping. Alignment of trimmed bisulfite‐converted sequences was carried out using Bismark (Babraham Institute, Cambridge, UK) [39] against the human reference genome for chromosome 5 (GRCh38, release 93), yielding methylation call percentages for each CpG and non‐CpG site within the chromosome. Duplicate reads arising from artifacts in library preparation and sequencing were deduplicated using Bismark, and Bismark methylation extractor was used to generate postalignment methylation counts. Aligned reads and methylation were visualized using Integrated Genomics Viewer (Broad Institute and UC San Diego, La Jolla, CA, USA). Graphs were prepared using prism 8 (GraphPad, version 8.4.0).

## 3 Results

### 3.1 Upstream *TERT* promoter hypermethylation and proximal promoter hypomethylation conserved across cancer tissue types

**Fig. 1:**
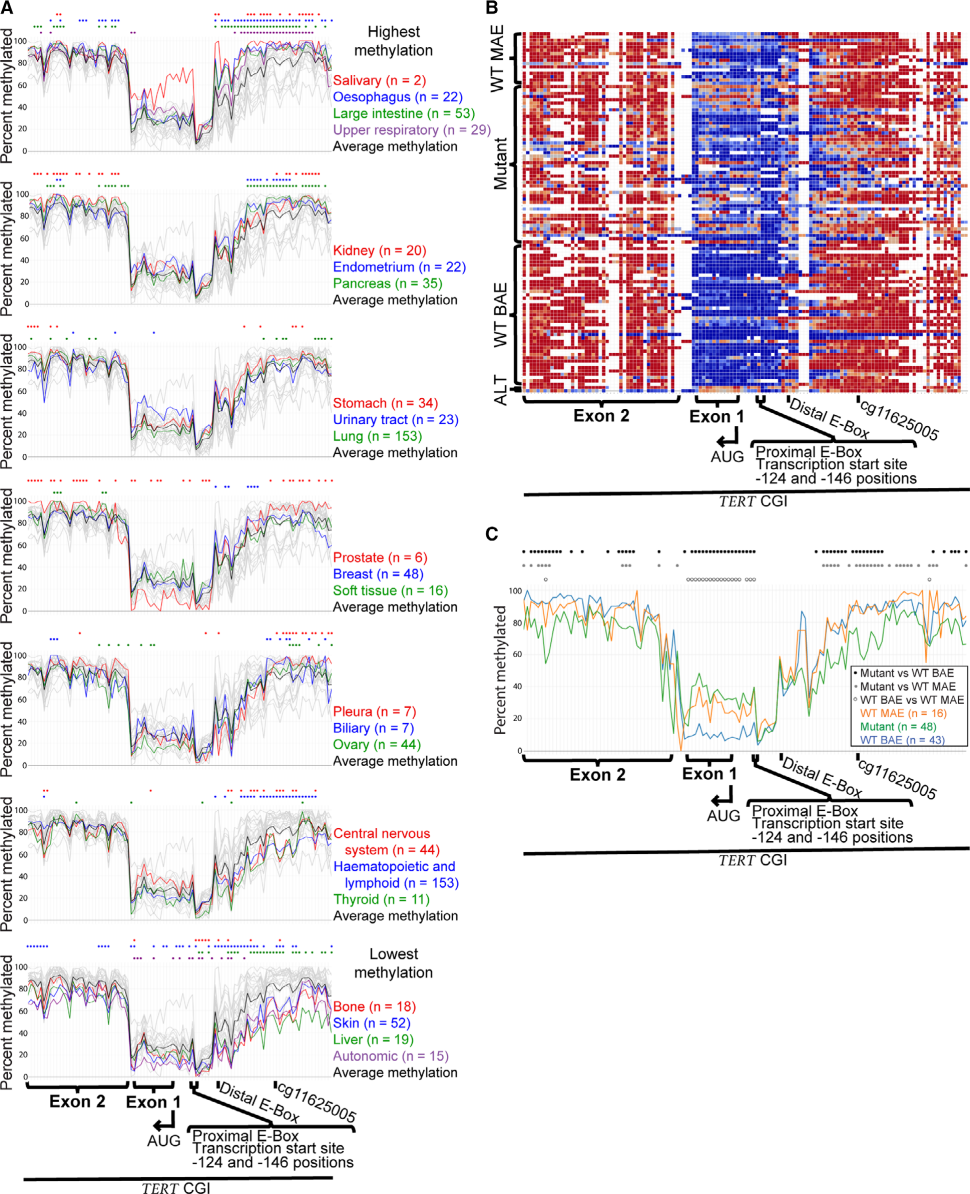
*TERT* promoter is characterized by conserved upstream hypermethylation and proximal hypomethylation across different cancer tissue types. (A) Bisulfite conversion sequencing (Bis‐Seq) DNA CpG methylation data for 95 positions across the *TERT* promoter for 23 different cancerous tissues, showing mean values from 833 cancer cell lines (*n* represents the number of cell lines per tissue). Colored circles indicate individual CpG sites with statistically significant (*P* ≤ 0.005) differences between the tissue and all other tissues. Each graph groups tissues by total mean percent methylated, from most to least methylated (top to bottom, respectively). Each chromosomal position includes data from at least two cell lines for all 23 tissues. (B) Bis‐Seq DNA CpG methylation data for 129 positions across the *TERT* promoter for 109 cell lines with known allelic expression and activating mutation classifications. Lines had been classified as having wild‐type (WT) monoallelic expression (MAE) of *TERT* (‘WT MAE’), −124 or −146 C>T activating promoter mutations (‘mutant’), biallelic expression (BAE) of *TERT* (‘WT BAE’), or alternative lengthening of telomeres (ALT). Each row represents a different cell line. Colors range from red to blue for more to less methylated CpGs, respectively. White represents unavailable data. Each chromosomal position includes data from at least 10 cell lines. (C) Bis‐Seq DNA CpG methylation data for 122 positions across the *TERT* promoter for 107 cell lines shown in 1B (*n* represents the number of cell lines per tissue). Colored circles indicate statistically significant (*P* ≤ 0.05) differences in the listed pairwise comparisons, where statistical analysis was performed using 2‐tailed Student's *t*‐test with unequal variance. Each chromosomal position includes data from at least two cell lines for all three cell types. See Table S1 for chromosomal positions and Table S2 for cell line data.

Using publicly available bisulfite conversion sequencing (Bis‐Seq) data (from the Broad Institute's Cancer Cell Line Encyclopedia [CCLE]: www.broadinstitute.org/ccle), we compared *TERT* promoter CpG methylation across 23 different cancer tissue types and 833 cancer cell lines. The comparison spanned a 2788‐bp region containing the upstream promoter through exon 2 (5:1296377–1293589; hg19 genomic coordinates) (Table S1). The mean methylation across this region varied for different tissue types from 46.4% to 78.9%, with 91% of tissues (21/23) being overall hypermethylated (> 50% methylated) (Fig. 1A). Values for each tissue are as shown in Table 1 (for individual cell line data, see Fig. S1 and Table S2).

**Table 1:** *TERT* promoter methylation values for different tissue types from Fig. 1A. *n*, number of cell lines analyzed

Tissue type	Mean percent methylated
Salivary	78.9 (*n* = 2)
Esophagus	73.3 (*n* = 22)
Large intestine	71.5 (*n* = 53)
Upper respiratory	69.8 (*n* = 29)
Kidney	69.6 (*n* = 20)
Endometrium	69.1 (*n* = 22)
Pancreas	68.6 (*n* = 35)
Stomach	68.5 (*n* = 34)
Urinary tract	65.4 (*n* = 23)
Lung	65.2 (*n* = 153)
Prostate	64.9 (*n* = 6)
Breast	64.6 (*n* = 48)
Soft tissue	64.6 (*n* = 16)
Pleura	63.7 (*n* = 7)
Biliary	63.2 (*n* = 7)
Ovary	59.8 (*n* = 44)
Central nervous system	59.7 (*n* = 44)
Haematopoietic and lymphoid	57.4 (*n* = 153)
Thyroid	55.0 (*n* = 11)
Bone	51.7 (*n* = 18)
Skin	50.5 (*n* = 52)
Liver	46.4 (*n* = 19)
Autonomic	46.4 (*n* = 15)

While significant variation was apparent between different tissue types, some conserved patterns of *TERT* methylation were observed (Fig. 1A). We describe these from right to left on the genome browser traces, because this is the direction in which *TERT* transcription occurs. A region of the upstream promoter (≈ 1296377–1295699) was highly methylated (52.4–94.5% methylated; mean = 82.5% for all tissues). This included a previously identified hypermethylated CpG biomarker (cg11625005; 1295737). A hyper‐ to hypomethylation transition occurred over a span of ≈ 347 bp, from ≈ 581 to 234 bp upstream of the translation start site (AUG) (≈ 1295685–1295338) (Fig. 1A). The distal E‐Box was within this region. The proximal promoter, which includes the promoter mutation sites, the somewhat heterogeneous transcription start site region, and the proximal E‐Box, contained the point of lowest methylation for nearly all tissue types (1295247, ≈ 143 bp upstream of AUG, between the −124 and −146 mutations). This point ranged from 0.4% to 11.2% methylated, with a mean value of 6.5% across all tissue types. Levels increased but remained hypomethylated through exon 1 (until 1294448) for nearly all tissue types (96%; 22/23); greater methylation levels in exon 1 were observed in some cell line types compared to others (see Section 3.2, below) (Fig. 1A). The transition from hypo‐ to hypermethylation occurred in intron 1, which contained data from relatively few CpGs (Fig. 1A); if data were available from additional CpG sites, this transition might appear more gradual. Exon 2 (≈ 1294441–1293589) was hypermethylated in all tissue types. This overall pattern is consistent with previous studies on more limited tissue types [16, 24, 26, 27]. Interestingly, in most tissues the hypermethylated upstream region showed significant variation between different tissue types, while the hypomethylated proximal promoter region was more constant.

To compare CpG methylation in normal adult cells and a human embryonic stem cell (hESC) line with some of the cancer cell lines, a limited analysis across the same region was performed using data from the Encyclopedia of DNA Elements (ENCODE) (Fig. S2, Table S1, Table S3). In contrast to the cancer cell lines, the cg11625005 biomarker and surrounding distal promoter region were hypomethylated in normal adult cells and hESCs (Fig. S2), in agreement with previous work [22]. Because hESCs express *TERT*, it seems unlikely that methylation and inactivation of repressor binding sites in this distal promoter region by themselves lead to *TERT* reactivation in cancer cells, as has been suggested [16, 24]. Hypomethylation of the proximal promoter–exon 1 region was similar between cancer and normal cells (Fig. S2). Because normal adult cells do not express *TERT*, hypomethylation of the proximal promoter is clearly insufficient, though potentially necessary, for gene expression.

### 3.2 *TERT* promoter methylation patterns associate with allelic expression classifications

*TERT* expression was analyzed in 107 cancer cell lines that were previously classified as follows: WT promoter, MAE (*n* = 16); −124 and −146 promoter mutations, which cause MAE (*n* = 48); and WT, BAE (*n* = 43) [9, 13]. Patterns of *TERT* methylation showed general similarity among cell lines in these categories (Fig. 1B,C). Mean methylation values were 68.2% for WT MAE, 66.7% for WT BAE, and 59.8% for mutants. Interestingly, some regions of the CGI did show methylation differences that associated with allelic expression and activating mutation classifications. Mutant lines displayed significantly decreased methylation compared to WT BAE and WT MAE lines (*P* < 0.05) across most of the upstream hypermethylated promoter region and part of the adjacent transition region (≈ 1296377–1295458), and at multiple positions in exon 2. In addition, WT BAE lines showed significantly decreased methylation relative to both WT and mutant lines across most of the hypomethylated proximal promoter region (≈ 1295139–1294873) (Fig. 1C). Specifically, this region of increased methylation in WT and mutant lines relative to WT BAE lines included exon 1, the AUG, and the proximal E‐Box. However, it did not include the more upstream elements of the transcription start site region and −124 and −146 sites, where low methylation levels were observed in all cell line types. This finding supports the conclusion that hypomethylation associates with transcriptional activity, because the WT BAE lines only have active *TERT* alleles, whereas the methylation data in MAE lines are an average of active and inactive alleles.

In addition, two lines analyzed were telomerase‐negative and used the alternative lengthening of telomeres (ALT) mechanism [40] (Table S2). ALT lines displayed relatively higher mean methylation values (70.2%), particularly across the transition region and adjacent hypomethylated proximal promoter. This is consistent with the conclusion that increased methylation of the proximal promoter associates with transcriptional inactivity.

### 3.3 Monoallelic expression of *TERT* correlates with allelic proximal promoter methylation

**Fig. 2:**
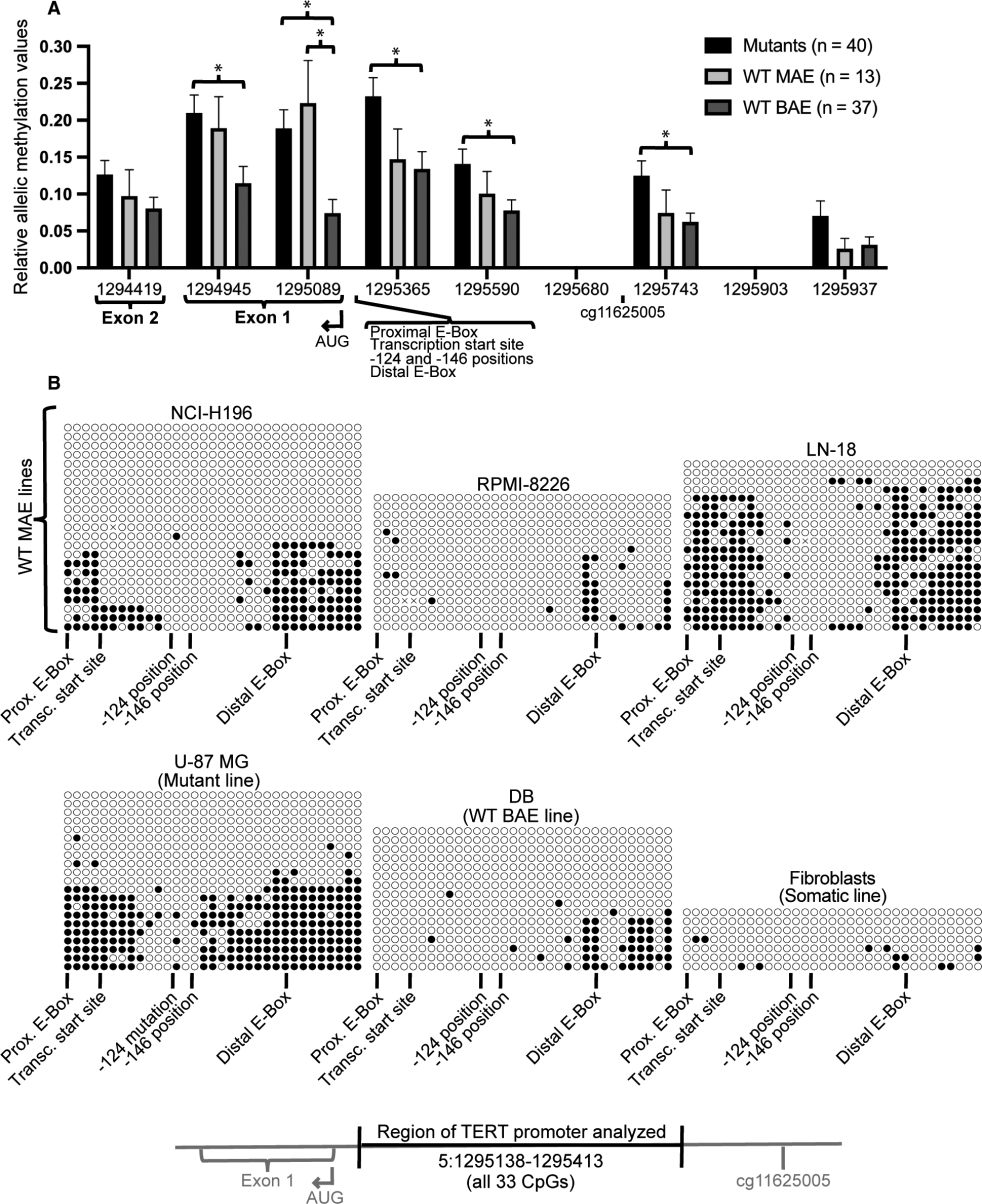
Cells with monoallelic expression of *TERT* have allelic methylation of the proximal *TERT* promoter. (A) Extent of allelic methylation across the *TERT* gene, which is transcribed from right to left. Relative allelic methylation was measured by calculating the difference between the mean and mode values of raw read Bis‐Seq CpG methylation data, where greater levels suggest greater allelic methylation behavior (see Fig. S3 for examples). Positions included contained 3–6 CpGs per read and coverage of ≥ 5 reads per cell line. Error bars represent standard error of the mean. *n* represents the number of cancer cell lines. *P ≤ 0.05, where statistical analysis was performed using 2‐tailed Student's *t*‐test with unequal variance. (B) Bisulfite conversion cloning data from genomic DNA of select CpGs flanking the *TERT* transcription start site (5:1295138–1295413, spanning 33 CpGs). Each row represents a different clone (or genome copy, or allele) and each circle represents a CpG, with black circles representing a methylated CpG and white circles representing an unmethylated CpG. For chromosomal positions of noted *TERT* features, see Table S1.

Using publicly available Bis‐Seq data (from CCLE), raw read analysis revealed allelic methylation behavior within the *TERT* proximal promoter and exon 1. Relative allelic methylation values were calculated based on the difference between the mean and mode of the percent methylation of reads at a given read position (Fig. S3). Using this calculation, the greater the difference between mean and mode, the more suggestive it is of allelic behavior. Averaged over all cell lines, the highest allelic values were in the hypomethylated proximal promoter region (1294945–1295363; Fig. 2A), including exon 1, the AUG, the transcription start site region, the −124 and −146 mutations, and both E‐Boxes. In this region and elsewhere, WT BAE lines displayed significantly lower allelic values compared to both WT MAE and mutant cell lines. This is consistent with the expectation that WT MAE and mutant cell lines have more allelic methylation than WT BAE lines.

To validate the apparent allelic methylation behavior, we performed bisulfite conversion cloning within the proximal *TERT* promoter (5:1295138–1295413; 33 CpGs total) (Fig. 2B). Bisulfite conversion cloning gives the methylation status of successive CpGs along single cloned copies of a gene, where each sequence must represent a single allele [41]. Each sequence is shown as a row of circles (with a white circle for an unmethylated CpG and black for a methylated CpG). The results aligned well with our Bis‐Seq analysis of this region (Fig. 1 and Fig. S4). Bis‐Seq analysis of WT MAE lines found the lowest methylation point to be the 5:1295247 read position (Fig. 1), and bisulfite conversion cloning showed the lowest methylation to be in the same region, ≈ 5:1295195–1295324. Also supporting the Bis‐Seq analysis, one WT MAE line (RPMI‐8226) had much lower overall methylation levels. The mutant line analyzed (U‐87 MG) contained a narrower hypomethylated area (≈ 5:1295189–1295260), also agreeing with the Bis‐Seq analysis for this cell line (Fig. S4). Furthermore, a WT BAE line (DB) had lower overall methylation, as we had seen for BAE lines in general, as well as for this specific line (Fig. 1B,C, and Fig. S4). Somatic cells (fibroblasts) had low levels of methylation throughout the region [0% (0/33) – 9% (3/33) of CpGs methylated per clone], consistent with earlier reports [22, 42].

In MAE lines, the CpGs flanking the hypomethylated region displayed allelic methylation behavior. Specifically, in this flanking region a given sequence was either entirely or mostly unmethylated (white circles in Fig. 2B), or the sequence was heavily methylated (black circles). Two distinct patterns of methylation are the expectation for allelic methylation. This was clearly seen in the U‐87 MG mutant line, where 43% of clones (9/21) had ≤ 6% of CpGs methylated and 48% (10/21) had 49% (16/33) – 79% (26/33) methylated CpGs. In the DB WT BAE line, methylation clustered on some alleles within an upstream region (≈ 5:1295341–1295413), potentially demonstrating allelic methylation here [56% (9/16) of clones contained no methylated CpGs, while 38% (6/16) contained 60% (6/10) – 80% (8/10) methylated CpGs]. Overall, allelic *TERT* methylation patterns were evident in most MAE lines analyzed, both *TERT* promoter mutant and WT.

### 3.4 Decreased methylation of the *TERT* promoter associates with histone marks of active transcription

**Fig. 3:**
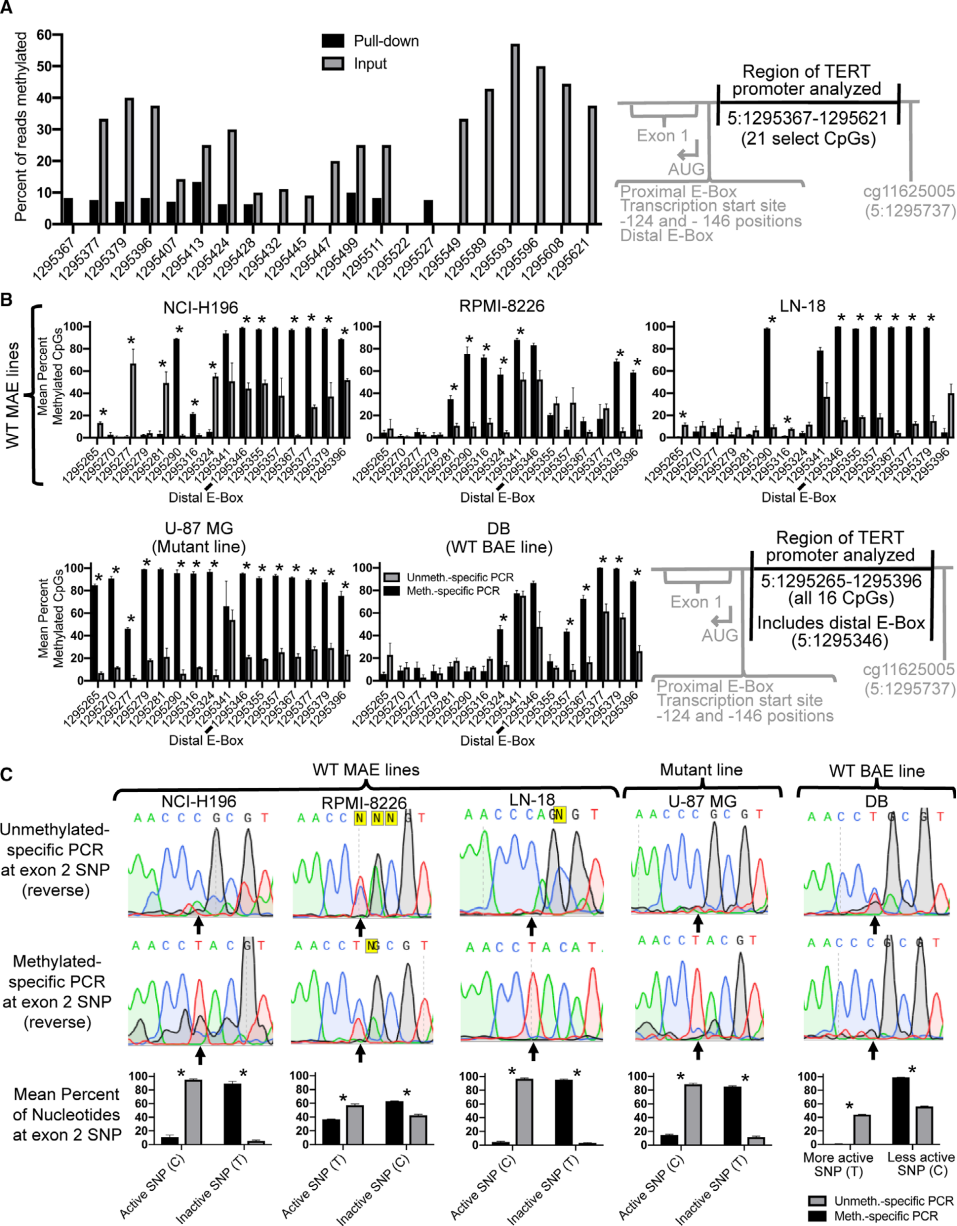
Decreased *TERT* promoter methylation associates with histone marks of active transcription and an active exonic SNP. (A) ChIP‐Bis‐Seq of the *TERT* promoter using an H3ac antibody shows enrichment of unmethylated DNA in the pulled‐down samples (black) relative to the input (gray) in LN‐18 cells. The absence of any bars indicates zero percent methylation. Inclusion criteria for read positions were a greater number of reads in the pull‐down relative to the input and ≥ 10 reads in the pull‐down (mean input coverage was 9 reads; mean pull‐down coverage was 13 reads; *P* = 0.01 for pull‐down efficiency). (B) Confirmation of long‐range bisulfite conversion PCR enriching for unmethylated or methylated CpGs at the *TERT* proximal promoter (16 CpGs spanning 5:1295265–1295396; region overlaps with some of the CpGs analyzed in 3A) using unmethylated (gray)‐ or methylated (black)‐specific bisulfite conversion PCR, respectively. PCR products generated a 1448‐bp product including the proximal promoter and the exon 2 SNP analyzed in Panel C. *P ≤ 0.05 (C) Long‐range bisulfite conversion PCR (same PCRs as shown in Panel B) showing representative Sanger sequencing results (upward arrow indicates position of the exon 2 SNP) and graphs of the sequencing results (*n* = 2–3 sequenced reactions). ‘Active SNP’ means that the nucleotide at the position of the SNP is the one found in the TERT mRNA transcribed in that cell line. The active SNP was either previously identified in all cell lines [14] or was identified here (Fig. S6). Error bars represent standard error of the mean. *P ≤ 0.01, where statistical analysis was performed using 2‐tailed Student's *t*‐test with unequal variance.

Using chromatin immunoprecipitation Bis‐Seq (ChIP‐Bis‐Seq), we found decreased *TERT* proximal promoter methylation to associate with increased histone marks of active transcription. The H3 acetylation (H3ac) antibody used binds acetylated lysine 9 (H3K9ac) and acetylated lysine 14 (H3K14ac) present together. This is tightly associated with active transcription start site regions and active genes [43]. ChIP using the H3ac antibody yielded efficient pull‐down and significant selection for the −124 mutant, active allele in the *TERT* promoter (Fig. S5). H3ac ChIP‐Bis‐Seq at the proximal *TERT* promoter had pull‐down of 21 CpGs spanning 5:1295250–1295504 (Fig. 3A). The majority of CpGs (18/21, or 86%) had decreased methylation in the H3ac‐pull‐down compared to the input (Fig. 3A). This indicates an association between decreased methylation and increased histone marks of active transcription in the *TERT* proximal promoter.

### 3.5 Decreased methylation of the *TERT* promoter associates with actively transcribed exon SNP

As an independent test of the relationship between *TERT* methylation and transcription, we performed long‐range PCR on bisulfite‐converted genomic DNA using unmethylated‐ or methylated‐specific primers. The resulting 1448 bp PCR product spanned the *TERT* proximal promoter and an exon 2 SNP (rs2736098, G/A) (see Table S4 for primer sequences). In genomic DNA containing a mixture of methylated and unmethylated alleles, unmethylated‐specific primers should amplify primarily unmethylated DNA, and methylated‐specific primers should amplify primarily methylated DNA. The exon 2 SNP was included to determine whether a primarily unmethylated or methylated product was associated with the active allele. We analyzed genomic DNA from cancer cell lines that were heterozygous for the *TERT* exon 2 SNP. The sequence of the active allele at this SNP was identified in all lines using RT‐PCR of RNA followed by Sanger sequencing of the PCR product [14] (Fig. S6). Additionally, in this way, lines with expression of only one allele were confirmed to have MAE; this included all WT MAE and mutant lines tested here. Lines with expression of both alleles were confirmed to have BAE of *TERT*; this included the WT BAE line tested here (Table S2 and Fig. S6) [14]. As a validation of the method, in the proximal *TERT* promoter all WT MAE and mutant lines had more methylated DNA in the methylated‐specific PCR products compared to the unmethylated‐specific products (Fig. 3B) [56.1 ± 1.0% vs. 30.9 ± 5.6% for NCI‐H196 (*P* = 0.07); 38.1 ± 3.4% vs. 16.6 ± 4.3% for RPMI‐8226 (*P* = 0.03); 50.0 ± 1.2% vs. 14.9 ± 3.9% for LN‐18 (*P* = 0.01); and 87.3 ± 2.8% vs. 19.1 ± 3.0% (*P* = 1 × 10^−14^) for U87].

This long‐range bisulfite conversion PCR approach confirmed associations between decreased *TERT* proximal promoter methylation and active *TERT* expression. In the same bisulfite‐converted PCR products, DNA sequencing was performed across exon 2, covering the position of the SNP. DNA sequencing of the exon 2 SNP showed significantly higher levels of the transcription‐associated SNP sequence in unmethylated‐specific products compared to methylated‐specific products (Fig. 3C; *P* ≤ 0.01). In agreement, in a WT BAE line that expressed one SNP nucleotide at relatively greater levels, the unmethylated‐specific PCR also significantly correlated with the more active nucleotide at the position of the SNP (*P* = 1 × 10^−5^). Overall, these long‐range bisulfite conversion PCR findings demonstrate an association between decreased *TERT* proximal promoter methylation and increased *TERT* expression.

## 4 Discussion

While the *TERT* promoter contains a strikingly hypermethylated distal promoter region in human cancer cells, here we found that hypomethylation at the proximal promoter, flanking the transcription start site region, is also strongly associated with active *TERT* transcription in cancer cells. This methylation pattern, and the proximal promoter hypomethylation in particular, was observed across 23 different cancerous tissue types, using Bis‐Seq data from 833 different cancer cell lines (Fig. 1A). In the hypomethylated proximal promoter region, cancer cell lines with apparent biallelic expression (BAE) of *TERT* had significantly lower methylation compared to lines with monoallelic expression (MAE) of *TERT*, consistent with decreased methylation here to be associated with *TERT* expression (Fig. 1B,C). In this proximal promoter region, methylation was allele‐specific (Fig. 2) and decreased methylation associated with marks of active TERT transcription (Fig. 3). Thus, *TERT* expression in cancer lines may be canonical after all, in terms of its association with low DNA CpG methylation.

The overall pattern of *TERT* promoter methylation is relatively conserved across a range of cancer tissue types, particularly in a proximal hypomethylated region, in alignment with previous studies [26]. While a *TERT* *‘*minimal promoter’ has been previously described, those studies typically focused on one or a few tissues and/or a handful of cancer cell lines per study, making it challenging to determine tissue‐specific differences and similarities. In particular, a ≈ 300 bp region of hypomethylation flanking the transcription start site region was previously labeled the ‘minimal promoter’, ranging roughly −260 to +40 bp of the translation start site (AUG) (5:1295364–1295064)[27]. Here, we provide resolution at the level of individual CpG sites in this hypomethylated region in *TERT*, which appears to be larger and slightly more downstream than previously described, spanning ≈ −220 to +231 bp of the AUG (or 5:1295321–1294873) (Fig. 1A). This includes all of *TERT* exon 1 and does not include the distal E‐Box site [16, 24]. This suggests that, in cancer cells, the more proximal E‐Box (5:1295138) may be more important for binding of methylation‐sensitive activating factors, such as c‐Myc/Max transcription factors [44, 45].

Hypomethylation of this *TERT* proximal promoter region may be a universal correlation and possibly a necessity for *TERT* expression in cancer cells, regardless of allelic expression status. We found this region to have significantly decreased methylation in *TERT* BAE cancer cells compared to MAE cells (Fig. 1B,C). Because WT BAE lines have only active *TERT* alleles, whereas MAE lines have both active and inactive alleles, this finding supports the idea that hypomethylation in this region is important for transcriptional activity. In this region, we also found decreased methylation to correlate with transcription in MAE cancer cells (Fig. 3). This agrees with previous findings showing active chromatin marks associated with unmethylated DNA in this region [16, 27]. Surprisingly, we saw similar, though less striking, allelic methylation correlations in apparently BAE cells, suggesting that even in BAE cells some allelic expression may occur and may depend upon decreased methylation of the more active alleles. Our group previously reported that in −124 mutant cells, active transcriptional histone marks (specifically H3K4me2/3) associate with decreased promoter methylation in the upstream hypermethylated region (1295619–1295738) [26]. It is also worth noting that human pluripotent stem cells, which express *TERT*, are largely hypomethylated across the *TERT* promoter [22]. Taken together, *TERT* activation appears canonically associated with decreased methylation in the proximal promoter. Ultimately, increasing methylation of the proximal *TERT* promoter to decrease *TERT* expression may be a route to explore for cancer therapeutics.

While we found the hypomethylated proximal *TERT* promoter region to contain apparently allelic methylation (Fig. 2), this should not be interpreted as one allele very lowly methylated and one allele with more methylation. Cancer lines often contain more than two *TERT* gene copies, with considerable line‐to‐line and cell‐to‐cell heterogeneity [14]. We previously performed *TERT* DNA FISH on several of the lines used here and found mean values of *TERT* DNA FISH spots per nucleus as 10.98 for NCI‐H196, 3.07 for LN‐18, 2.17 for U‐87 MG, and 3.88 for DB (RPMI‐8226 was not investigated). Hence, because in these cells there are multiple *TERT* gene copies, one or more of which may be inactive, drawing precise conclusions about allelic methylation patterns is challenging.

Considering the hypermethylation of the distal *TERT* promoter around cg11625005, which serves as a biomarker for identification and prognosis of cancer cells, it could contribute positively to gene expression [46]. If so, then the hESCs, which have a methylation pattern resembling telomerase‐minus normal human cells, would be an exception. Alternatively, the presence of this hypermethylation may be associated with, but not causal of, the cancer cell state, possibly due to larger‐scale chromatin structure remodeling events that occur in cancer cells.

A limitation of our findings here is that our data are correlative and not causal. Previous studies have altered the DNA CpG methylation of the *TERT* promoter with varying and apparently contradictory effects on *TERT* expression (reviewed by [47]). These disparate results may be due to using approaches that altered methylation in a nontargeted manner or using exogenous *TERT* promoter constructs. Specifically, most of these studies used 5‐azacytidine or 5‐aza‐2′‐deoxycytidine (decitabine; DAC) treatment, which results in global genomic demethylation. In some studies using cancer cells, these treatments caused decreased *TERT* expression, which may actually be due to demethylation of a downstream CTCF repressor binding site [28, 42, 48]. However, senescing fibroblasts, in which the *TERT* promoter had become hypermethylated, gained increased *TERT* expression upon promoter demethylation via DAC treatment [49]. An area of future investigation would be to alter DNA methylation in the *TERT* promoter in a targeted manner. For example, dCas9 techniques that tether DNMT3A to increase DNA methylation [50] could be targeted to the hypomethylated proximal promoter region; decreased *TERT* expression would then be predicted based on our findings here. Using a dCas9 technique tethering TET1 to locally decrease DNA methylation [51] in this region may not significantly increase *TERT* expression since this region is already hypomethylated.

Overall, our findings that hypomethylation of the proximal *TERT* promoter is shared across a range of cancer tissue types and that decreased methylation is associated with increased transcription regardless of cancer cell line type suggest that this hypomethylated area may be necessary for active *TERT* expression. However, because the proximal promoter region is also hypomethylated in somatic cells that do not express *TERT*, it is clearly not sufficient for *TERT* expression. Yet, the possibility of an underlying *TERT* activation mechanism that is necessary, specific, and universal to cancer cells is noteworthy. While −124 mutants are known to have a mutation that recruits ETS transcription factors such as GABP to activate *TERT* expression [10], still approximately 78% of cancer cell lines have wild‐type *TERT* promoters and do not contain known activating *cis‐*acting genetic alterations [9]. This makes it unclear how the majority of cancer lines have active *TERT* expression. It may be that *TERT* expression is activated by aberrant upregulation of activating transcription factors in cancer cells that recognize binding motifs in this hypomethylated region, such as c‐Myc/Max binding of the proximal *TERT* promoter E‐Box. Future studies may investigate whether targeted methylation or other inhibition of the proximal E‐Box results in decreased *TERT* expression in cancer cells. If this is found to be the case, such targeted approaches may be useful to explore for potential cancer therapeutics.

## 5 Conclusion

Across 23 cancerous tissue types, we found DNA hypermethylation in the upstream *TERT* promoter region and relatively more conserved hypomethylation of a proximal promoter region and exon 1. This region showed allelic methylation in cancer lines with MAE of *TERT*. Decreased methylation of the proximal *TERT* promoter is associated with, and may be important for, active *TERT* expression in cancer cells.

## Conflict of interest

TRC is on the board of directors of Merck and Co., Inc., and an advisor to Storm Therapeutics and Eikon Therapeutics.

## Author contributions

TJR and TRC conceived the project and designed the research. TJR designed the experiments. TJR, AJB, and TRC analyzed the data. TJR and TRC wrote the manuscript.

## Supporting information


**Fig. S1.** *TERT* promoter consistently has upstream hypermethylation and proximal hypomethylation across different cancer tissue types. Bis‐Seq DNA CpG methylation data for 129 positions across the *TERT* promoter (same positions as shown in Fig. 1B) for all 23 tissues and 833 cell lines (also analyzed in Fig. 1A). 109 cell lines had been classified as having wildtype (WT) monoallelic expression (MAE) of TERT (“WT MAE”), ‐124 or ‐146 C>T activating promoter mutations (“mutant”), biallelic expression (BAE) of TERT (“WT BAE”), or alternative lengthening of telomeres (ALT). All other cell lines either do not belong to one of these categories or were unknown to be classified for this analysis (“Unclassified”). Each row represents a different cell line. Colors range from red to blue for more to less methylated CpGs, respectively. White represents unavailable data. See Table S1 for chromosomal positions and Table S2 for cell line data.

**Fig. S2.** *TERT* promoter CpG methylation data in select cancer cell lines, normal adult cells, and hESCs from the ENCODE UCSC Genome Browser. DNA CpG methylation data for 151 positions across the *TERT* promoter (spanning the same region shown in Fig. 1 and Fig. S1) for 12 cancer cell lines (all cancer lines are also present in Fig. S1), 6 normal adult cell lines, and 1 hESC line. Each row represents a different cell line. Colors range from red to blue for more to less methylated CpGs, respectively. White represents unavailable data. See Table S1 for chromosomal positions and Table S3 for cell line data.

**Fig. S3.** Examples of bisulfite conversion sequencing (Bis‐Seq) raw read analysis (CCLE Bis‐Seq dataset). Each graph shows an example analysis of Bis‐Seq reads for a single cancer cell line, at a single CpG position, to determine potentially different degrees of allelic methylation (used in Figure 2A). Graphs are shown in order of increasing likelihood of possessing allelic methylation (S5A‐S5D). Read positions included for analysis contained 3 ‐ 6 CpGs per read and coverage of ≥5 reads per cell line. For calculations, methylated CpGs were assigned a value of 1 and unmethylated CpGs a value of 0. The greater the difference between the mean and mode calculations, the more suggestive it is of allelic methylation behavior.

**Fig. S4.** Select cell line data from Figure 1A showing bisulfite conversion sequencing (Bis‐Seq) data (CCLE Bis‐Seq dataset) for cell lines used in Fig. 2B, 3B, and 3C. CCLE Bis‐Seq data was unavailable for line LN‐18.

**Fig. S5.** Chromatin immunoprecipitation (ChIP) validation of H3ac antibody that was used in ChIP bisulfite sequencing (ChIP‐Bis‐Seq). (A) ChIP using H3ac antibody showed effective pull‐down at the proximal *TERT* promoter, with 2.6‐4.0% of input pulled down (for primers used, see Table S4). (B) ChIP pull‐down in this same region demonstrated significant enrichment for the active, mutant ‐124 allele in two different ‐124 mutant cell lines (U‐87 MG and SK‐N‐SH). Error bars represent standard error of the mean (SEM). *p≤0.02, where statistical analysis was performed using 2‐tailed Student's t‐test with unequal variance.

**Fig. S6.** Identification of the active *TERT* SNP in Exon 2. Genomic DNA (gDNA) and Reverse‐Transcription PCR (RT‐PCR) sequencing of *TERT* exon 2 SNP in RPMI‐8226 cells (WT MAE for *TERT*). All other cell lines shown in Fig. 3B and 3C had been previously analyzed in this manner to identify the active exon 2 *TERT* SNP [14].

**Table S1.** *TERT* promoter features and positions, including specific CpGs, interrogated in Figures 1A, 1B, and 2B, and Supplemental Figures 1 and 2.

**Table S2.** Cell line and tissue Bis‐Seq DNA CpG methylation values from 833 cell lines and 23 different cancerous tissue types shown in Figure 1 and Supplemental Figure 1, where methylated CpGs receive a value of 1 and unmethylated CpGs receive a 0.

**Table S3.** Cell line DNA CpG methylation values from 12 cancer cell lines, 6 normal adult cell lines, and 1 hESC line from the ENCODE UCSC Genome Browser shown in Fig. S2, where methylated CpGs receive a value of 1 and unmethylated CpGs receive a 0.

**Table S4.** Primer sequences for Figures 2B, 3B, 3C and Figures S5, S6.
