# Supplementary figures and images for: Allele‐specific proximal promoter hypomethylation of the telomerase reverse transcriptase gene (TERT) associates with TERT expression in multiple cancers
Source: Mol Oncol. 2020 Sep 11;14(10):2358–74. doi: 10.1002/1878-0261.12786 (PMC7530785; doi:10.1002/1878-0261.12786)

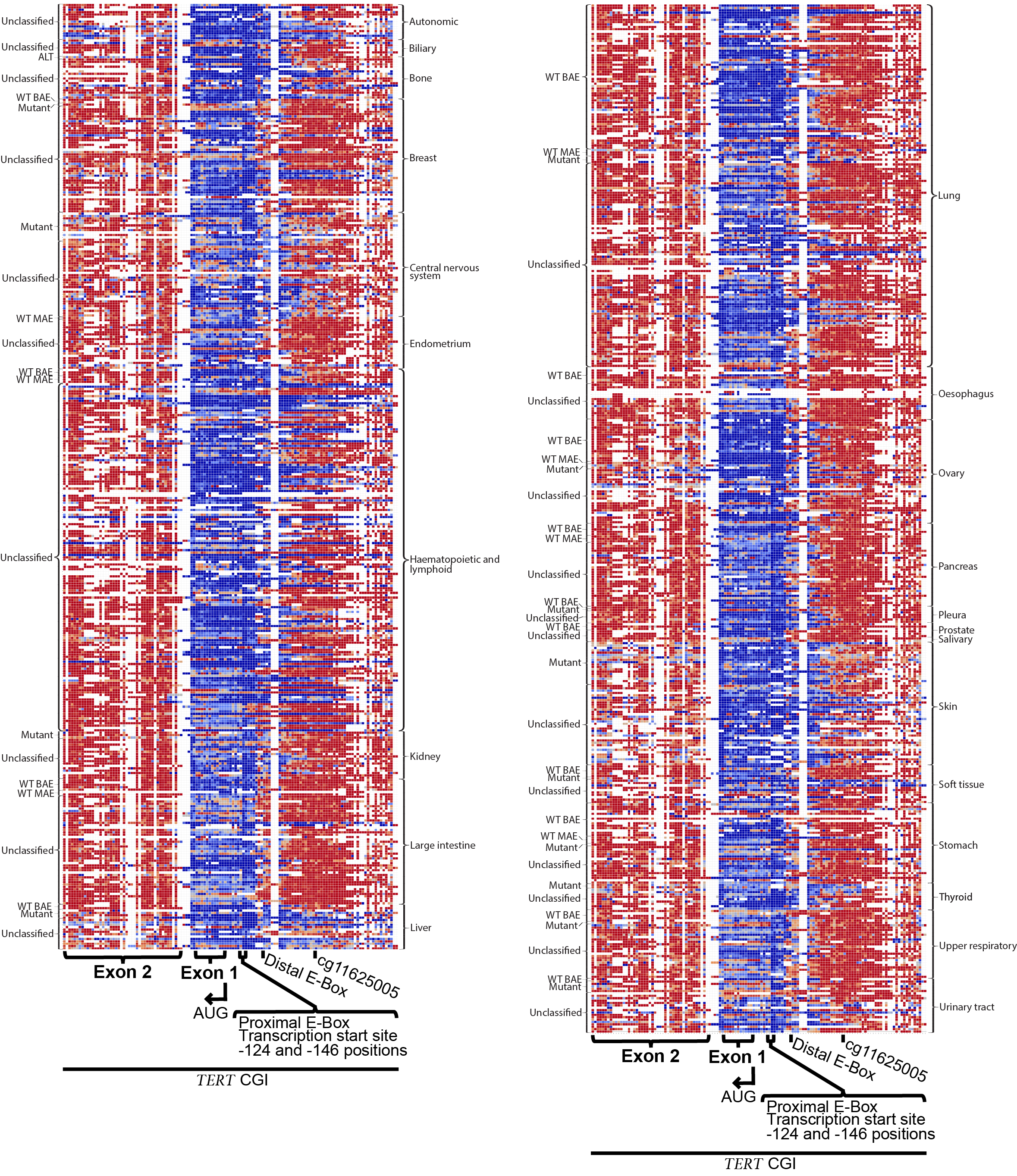

Supplement: Supplementary file 1 — Fig. S1. TERT promoter consistently has upstream hypermethylation and proximal hypomethylation across different cancer tissue types. Bis‐Seq DNA CpG methylation data for 129 positions across the TERT promoter (same positions as shown in Fig. 1B) for all 23 tissues and 833 cell lines (also analyzed in Fig. 1A). 109 cell lines had been classified as having wildtype (WT) monoallelic expression (MAE) of TERT (“WT MAE”), ‐124 or ‐146 C>T activating promoter mutations (“mutant”), biallelic expression (BAE) of TERT (“WT BAE”), or alternative lengthening of telomeres (ALT). All other cell lines either do not belong to one of these categories or were unknown to be classified for this analysis (“Unclassified”). Each row represents a different cell line. Colors range from red to blue for more to less methylated CpGs, respectively. White represents unavailable data. See Table S1 for chromosomal positions and Table S2 for cell line data. [file MOL2-14-2358-s001.tif]

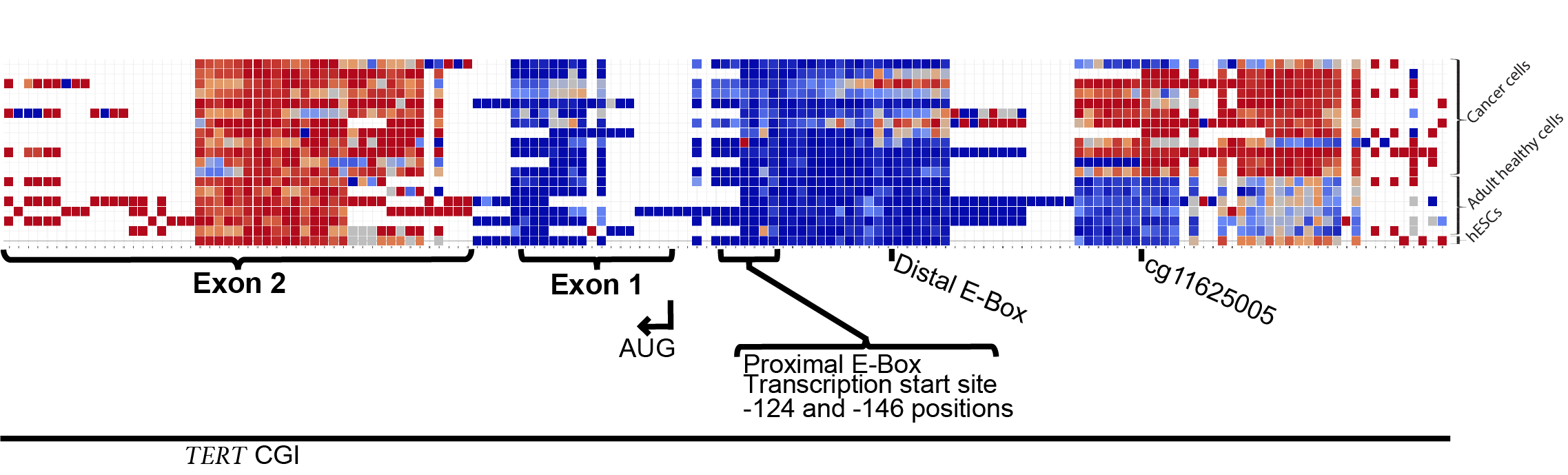

Supplement: Supplementary file 2 — Fig. S2. TERT promoter CpG methylation data in select cancer cell lines, normal adult cells, and hESCs from the ENCODE UCSC Genome Browser. DNA CpG methylation data for 151 positions across the TERT promoter (spanning the same region shown in Fig. 1 and Fig. S1) for 12 cancer cell lines (all cancer lines are also present in Fig. S1), 6 normal adult cell lines, and 1 hESC line. Each row represents a different cell line. Colors range from red to blue for more to less methylated CpGs, respectively. White represents unavailable data. See Table S1 for chromosomal positions and Table S3 for cell line data. [file MOL2-14-2358-s002.tif]

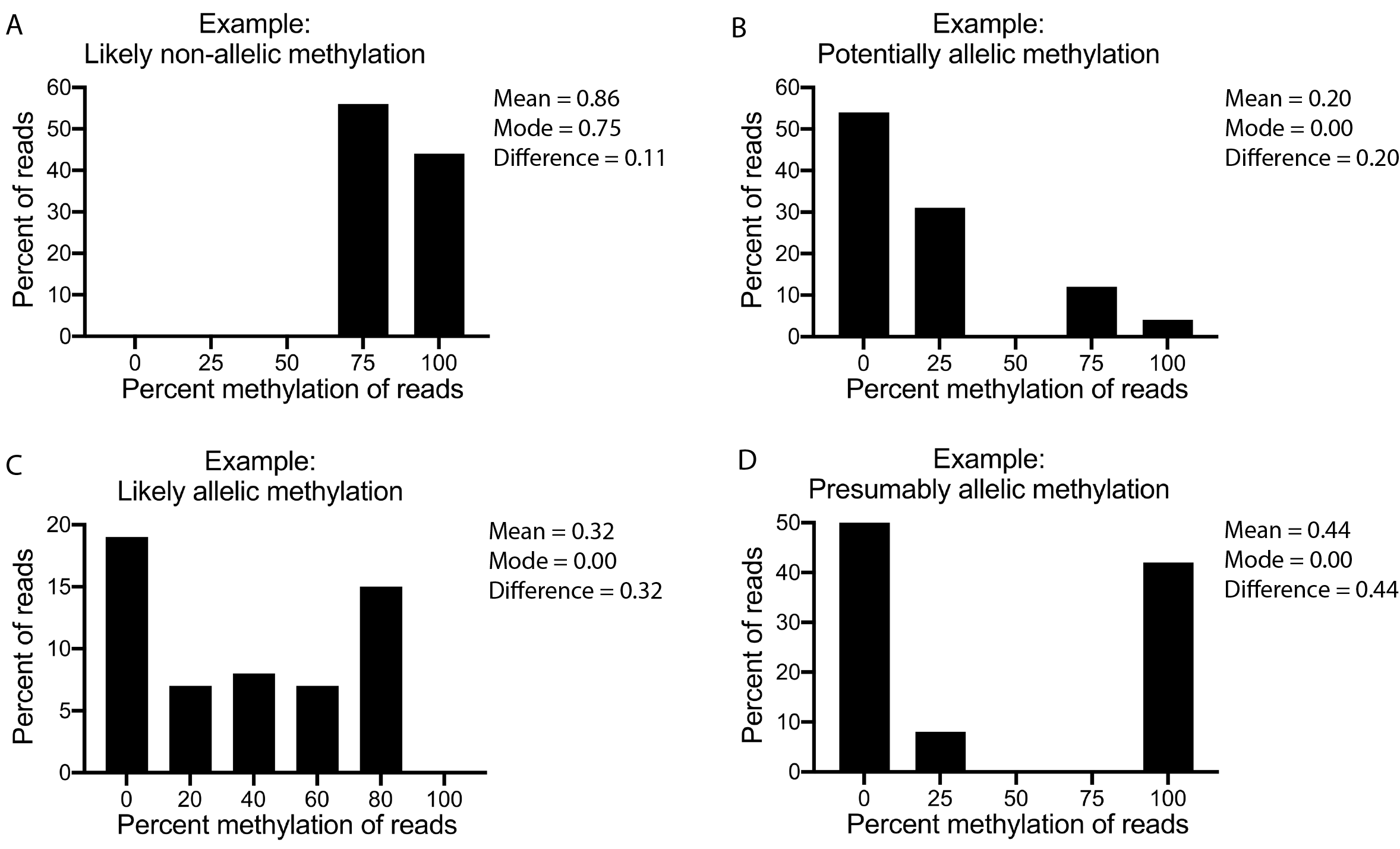

Supplement: Supplementary file 3 — Fig. S3. Examples of bisulfite conversion sequencing (Bis‐Seq) raw read analysis (CCLE Bis‐Seq dataset). Each graph shows an example analysis of Bis‐Seq reads for a single cancer cell line, at a single CpG position, to determine potentially different degrees of allelic methylation (used in Figure 2A). Graphs are shown in order of increasing likelihood of possessing allelic methylation (S5A‐S5D). Read positions included for analysis contained 3 ‐ 6 CpGs per read and coverage of ≥5 reads per cell line. For calculations, methylated CpGs were assigned a value of 1 and unmethylated CpGs a value of 0. The greater the difference between the mean and mode calculations, the more suggestive it is of allelic methylation behavior. [file MOL2-14-2358-s003.tif]

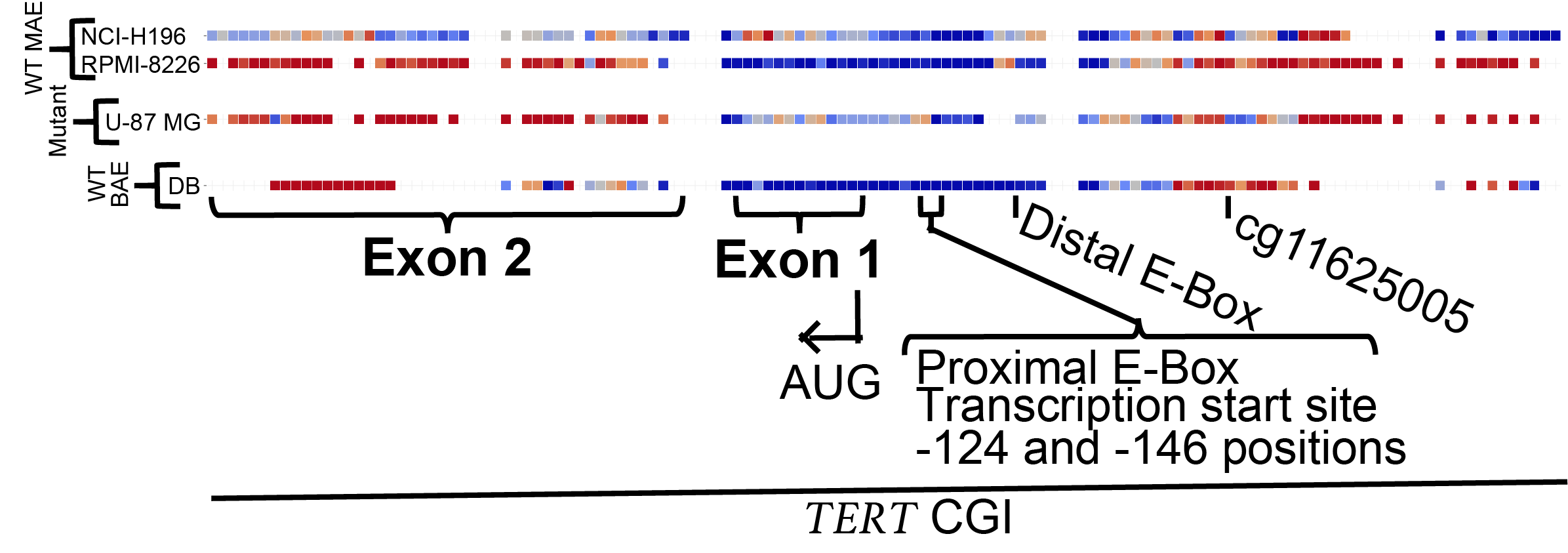

Supplement: Supplementary file 4 — Fig. S4. Select cell line data from Figure 1A showing bisulfite conversion sequencing (Bis‐Seq) data (CCLE Bis‐Seq dataset) for cell lines used in Fig. 2B, 3B, and 3C. CCLE Bis‐Seq data was unavailable for line LN‐18. [file MOL2-14-2358-s004.tif]

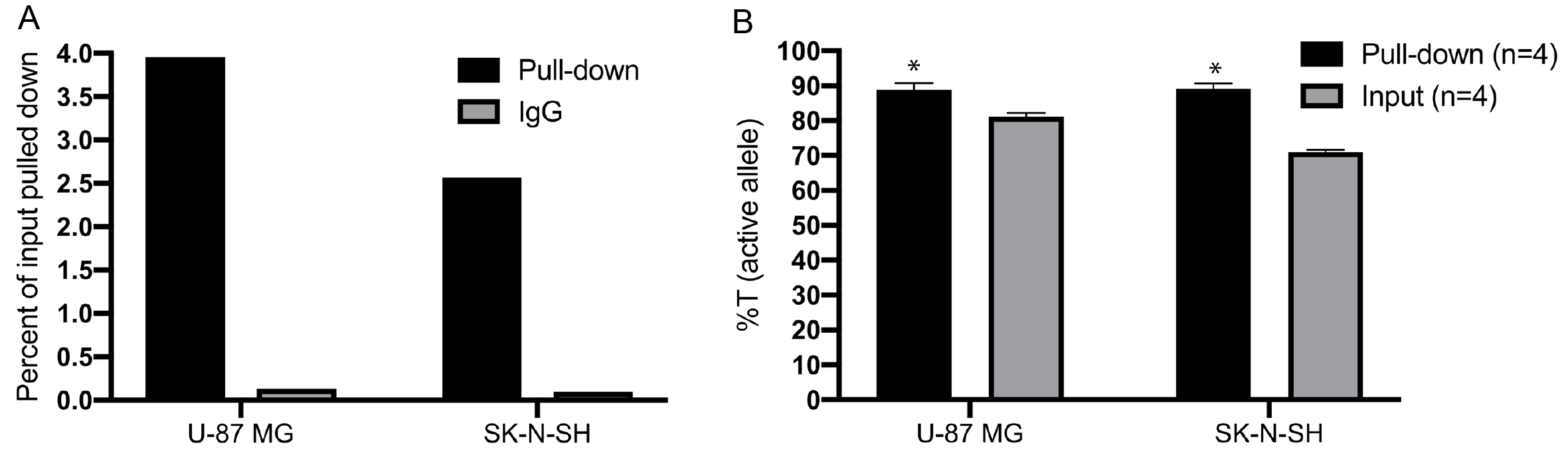

Supplement: Supplementary file 5 — Fig. S5. Chromatin immunoprecipitation (ChIP) validation of H3ac antibody that was used in ChIP bisulfite sequencing (ChIP‐Bis‐Seq). (A) ChIP using H3ac antibody showed effective pull‐down at the proximal TERT promoter, with 2.6‐4.0% of input pulled down (for primers used, see Table S4). (B) ChIP pull‐down in this same region demonstrated significant enrichment for the active, mutant ‐124 allele in two different ‐124 mutant cell lines (U‐87 MG and SK‐N‐SH). Error bars represent standard error of the mean (SEM). *p≤0.02, where statistical analysis was performed using 2‐tailed Student's t‐test with unequal variance. [file MOL2-14-2358-s005.tif]

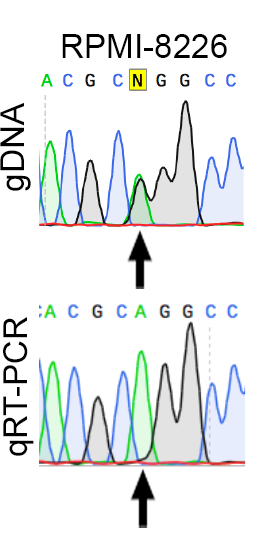

Supplement: Supplementary file 6 — Fig. S6. Identification of the active TERT SNP in Exon 2. Genomic DNA (gDNA) and Reverse‐Transcription PCR (RT‐PCR) sequencing of TERT exon 2 SNP in RPMI‐8226 cells (WT MAE for TERT). All other cell lines shown in Fig. 3B and 3C had been previously analyzed in this manner to identify the active exon 2 TERT SNP [14]. [file MOL2-14-2358-s006.tif]
